# Supplementary material for: Gut bacterial communities in roadkill animals: A pioneering study of two species in the Amazon region in Ecuador
Source: PLoS One. 2024 Dec 30;19(12):e0313263. doi: 10.1371/journal.pone.0313263 (PMC11684718; doi:10.1371/journal.pone.0313263)
Supplement: S1 Table — (DOCX) [file pone.0313263.s003.docx]

**Table S1. Sequencing data from the analyses of two vertebrate species for this study.**

| **Sample** | **Species** | **Estimated time since death** | **Total Reads** | **Total Data in Mb** |
| --- | --- | --- | --- | --- |
| SW001 | *Amphisbaena bassleri* | 0 hours | 153,136 | 41.2 |
| SW002 | *Amphisbaena bassleri* | 0 hours | 268,832 | 71.4 |
| SW003 | *Amphisbaena bassleri* | 2 hours | 69,538 | 17.4 |
| SW004 | *Amphisbaena bassleri* | 6 hours | 242,662 | 57.8 |
| SW005 | *Crotophaga ani* | 1 hours | 518,250 | 139.0 |
| SW006 | *Crotophaga ani* | 1 hours | 248,648 | 63.0 |
| SW007 | *Crotophaga ani* | 2 hours | 238,112 | 62.4 |
| SW008 | *Crotophaga ani* | 6 hours | 340,430 | 91.2 |
| SW009 | *Crotophaga ani* | 48 hours | 121,826 | 32.8 |
