# Supplementary material for: Gut bacterial communities in roadkill animals: A pioneering study of two species in the Amazon region in Ecuador
Source: PLoS One. 2024 Dec 30;19(12):e0313263. doi: 10.1371/journal.pone.0313263 (PMC11684718; doi:10.1371/journal.pone.0313263)
Supplement: S2 Table — (DOCX) [file pone.0313263.s004.docx]

**Table S2. Relative abundance at the phylum level in *A. bassleri* gut samples.**

| **Sample** | **Estimated time since death** | **Phylum** | **Relative Abundance (%)** |
| --- | --- | --- | --- |
| SW001 | 0 hours | Bacteroidetes | 0.75 |
| SW001 | 0 hours | Cyanobacteria | 0.01 |
| SW001 | 0 hours | Firmicutes | 95.42 |
| SW001 | 0 hours | Others | 0.95 |
| SW001 | 0 hours | Proteobacteria | 0.17 |
| SW001 | 0 hours | Verrucomicrobia | 2.69 |
| SW002 | 0 hours | Bacteroidetes | 0.22 |
| SW002 | 0 hours | Cyanobacteria | 0.07 |
| SW002 | 0 hours | Firmicutes | 98.13 |
| SW002 | 0 hours | Others | 0.87 |
| SW002 | 0 hours | Proteobacteria | 0.70 |
| SW002 | 0 hours | Verrucomicrobia | 0.00 |
| SW003 | 2 hours | Bacteroidetes | 1.84 |
| SW003 | 2 hours | Cyanobacteria | 0.05 |
| SW003 | 2 hours | Firmicutes | 69.51 |
| SW003 | 2 hours | Others | 1.30 |
| SW003 | 2 hours | Proteobacteria | 1.53 |
| SW003 | 2 hours | Verrucomicrobia | 25.77 |
| SW004 | 6 hours | Bacteroidetes | 5.87 |
| SW004 | 6 hours | Cyanobacteria | 4.70 |
| SW004 | 6 hours | Firmicutes | 78.73 |
| SW004 | 6 hours | Others | 5.25 |
| SW004 | 6 hours | Proteobacteria | 4.89 |
| SW004 | 6 hours | Verrucomicrobia | 0.56 |

The 'Others' category corresponds to all phyla that showed a relative abundance of less than 3% in at least one sample.
