# Supplementary material for: Gut bacterial communities in roadkill animals: A pioneering study of two species in the Amazon region in Ecuador
Source: PLoS One. 2024 Dec 30;19(12):e0313263. doi: 10.1371/journal.pone.0313263 (PMC11684718; doi:10.1371/journal.pone.0313263)
Supplement: S3 Table — (DOCX) [file pone.0313263.s005.docx]

**Table S3. The 25 most abundant families in *A. bassleri* gut samples.**

| **Family** | **Sample SW001** | **Sample SW002** | **Sample SW003** | **Sample SW004** |
| --- | --- | --- | --- | --- |
| Akkermansiaceae | 7.24 | 1.10 | 8.81 | 5.03 |
| Bacillaceae | 4.94 | 4.30 | 5.12 | 5.14 |
| Bacteroidaceae | 4.95 | 3.18 | 4.84 | 6.41 |
| Unclassified Bacteroidales | 4.11 | 0.69 | 3.00 | 4.28 |
| Burkholderiaceae | 0.69 | 4.67 | 2.40 | 5.69 |
| Christensenellaceae | 6.82 | 8.21 | 6.23 | 5.35 |
| Chroococcidiopsaceae | 0.00 | 2.83 | 2.08 | 6.99 |
| Clostridiaceae 1 | 9.23 | 10.08 | 8.04 | 8.86 |
| Unclassified Clostridiales | 3.61 | 5.58 | 3.81 | 4.44 |
| Clostridiales vadinBB60 group | 3.33 | 3.09 | 1.95 | 3.14 |
| Desulfovibrionaceae | 3.71 | 2.40 | 3.26 | 4.50 |
| Eggerthellaceae | 6.02 | 5.88 | 5.19 | 5.69 |
| Enterobacteriaceae | 3.18 | 4.39 | 5.82 | 5.10 |
| Erysipelotrichaceae | 7.86 | 8.32 | 6.72 | 7.51 |
| Family XIII | 6.99 | 6.90 | 6.75 | 6.89 |
| Lachnospiraceae | 9.15 | 8.90 | 8.95 | 8.05 |
| Mollicutes RF39 fa | 3.53 | 0.00 | 4.80 | 3.91 |
| Peptococcaceae | 5.63 | 5.06 | 4.51 | 5.34 |
| Peptostreptococcaceae | 9.89 | 10.30 | 7.93 | 8.51 |
| Prevotellaceae | 0.00 | 4.16 | 1.10 | 5.07 |
| Pseudomonadaceae | 1.61 | 4.19 | 1.10 | 5.16 |
| Rikenellaceae | 4.75 | 3.64 | 5.53 | 6.16 |
| Ruminococcaceae | 8.48 | 6.68 | 7.61 | 7.72 |
| Tannerellaceae | 3.87 | 2.48 | 4.20 | 5.42 |
| Uncultured Bacteria | 1.61 | 3.30 | 1.39 | 4.04 |
| Veillonellaceae | 2.77 | 3.47 | 4.26 | 3.95 |

The values for each sample correspond to the logarithmic transformation: log(x + 1), where x represents the total number of OTUs for that family in the sample.
