# Supplementary material for: Gut bacterial communities in roadkill animals: A pioneering study of two species in the Amazon region in Ecuador
Source: PLoS One. 2024 Dec 30;19(12):e0313263. doi: 10.1371/journal.pone.0313263 (PMC11684718; doi:10.1371/journal.pone.0313263)
Supplement: S4 Table — (DOCX) [file pone.0313263.s006.docx]

**Table S4. Relative abundance at the phylum level in *C. ani* gut samples.**

| **Sample** | **Estimated time since death** | **Phylum** | **Relative Abundance (%)** |
| --- | --- | --- | --- |
| SW005 | 1 hours | Actinobacteria | 25.34 |
| SW005 | 1 hours | Bacteroidetes | 0.23 |
| SW005 | 1 hours | Chlamydiae | 5.02 |
| SW005 | 1 hours | Epsilonbacteraeota | 0.19 |
| SW005 | 1 hours | Firmicutes | 68.33 |
| SW005 | 1 hours | Others | 0.35 |
| SW005 | 1 hours | Proteobacteria | 0.54 |
| SW006 | 1 hours | Actinobacteria | 0.72 |
| SW006 | 1 hours | Bacteroidetes | 0.00 |
| SW006 | 1 hours | Chlamydiae | 0.00 |
| SW006 | 1 hours | Epsilonbacteraeota | 0.00 |
| SW006 | 1 hours | Firmicutes | 97.90 |
| SW006 | 1 hours | Others | 0.15 |
| SW006 | 1 hours | Proteobacteria | 1.24 |
| SW007 | 2 hours | Actinobacteria | 12.80 |
| SW007 | 2 hours | Bacteroidetes | 0.57 |
| SW007 | 2 hours | Chlamydiae | 0.00 |
| SW007 | 2 hours | Epsilonbacteraeota | 18.83 |
| SW007 | 2 hours | Firmicutes | 50.23 |
| SW007 | 2 hours | Others | 1.03 |
| SW007 | 2 hours | Proteobacteria | 16.53 |
| SW008 | 6 hours | Actinobacteria | 17.52 |
| SW008 | 6 hours | Bacteroidetes | 0.85 |
| SW008 | 6 hours | Chlamydiae | 0.08 |
| SW008 | 6 hours | Epsilonbacteraeota | 0.00 |
| SW008 | 6 hours | Firmicutes | 75.33 |
| SW008 | 6 hours | Others | 0.22 |
| SW008 | 6 hours | Proteobacteria | 6.01 |
| SW009 | 48 hours | Actinobacteria | 20.08 |
| SW009 | 48 hours | Bacteroidetes | 3.27 |
| SW009 | 48 hours | Chlamydiae | 0.00 |
| SW009 | 48 hours | Epsilonbacteraeota | 0.00 |
| SW009 | 48 hours | Firmicutes | 74.75 |
| SW009 | 48 hours | Others | 0.98 |
| SW009 | 48 hours | Proteobacteria | 0.92 |

The 'Others' category corresponds to all phyla that showed a relative abundance of less than 3% in at least one sample.
