# Supplementary material for: Gut bacterial communities in roadkill animals: A pioneering study of two species in the Amazon region in Ecuador
Source: PLoS One. 2024 Dec 30;19(12):e0313263. doi: 10.1371/journal.pone.0313263 (PMC11684718; doi:10.1371/journal.pone.0313263)
Supplement: S5 Table — (DOCX) [file pone.0313263.s007.docx]

**Table S5. The 25 most abundant families in *C. ani* gut samples.**

| **Family** | **Sample SW005** | **Sample SW006** | **Sample SW007** | **Sample SW008** | **Sample SW009** |
| --- | --- | --- | --- | --- | --- |
| Atopobiaceae | 7.03 | 0.00 | 5.01 | 6.25 | 5.49 |
| Bacillaceae | 3.64 | 3.53 | 4.57 | 4.19 | 2.83 |
| Bacteroidaceae | 5.45 | 0.00 | 5.48 | 4.78 | 5.54 |
| Beijerinckiaceae | 4.54 | 4.85 | 6.65 | 5.89 | 2.83 |
| Burkholderiaceae | 4.50 | 4.45 | 5.32 | 3.37 | 4.23 |
| Clostridiaceae 1 | 3.30 | 2.48 | 4.68 | 4.81 | 2.71 |
| Coriobacteriaceae | 9.13 | 0.00 | 8.62 | 9.62 | 8.56 |
| Coriobacteriales Incertae Sedis | 10.21 | 0.00 | 0.00 | 4.17 | 3.95 |
| Unclassified Coriobacteriales | 6.44 | 0.00 | 5.44 | 6.05 | 4.97 |
| Corynebacteriaceae | 3.22 | 4.53 | 5.49 | 4.90 | 2.20 |
| Diplorickettsiaceae | 0.00 | 5.21 | 8.17 | 8.80 | 1.10 |
| Eggerthellaceae | 8.82 | 0.00 | 7.33 | 8.50 | 8.06 |
| Enterobacteriaceae | 4.73 | 4.89 | 8.38 | 4.83 | 3.43 |
| Enterococcaceae | 4.74 | 11.38 | 8.67 | 9.42 | 1.39 |
| Erysipelotrichaceae | 9.38 | 0.00 | 6.83 | 7.33 | 7.39 |
| Eubacteriaceae | 8.12 | 0.00 | 7.74 | 8.83 | 7.96 |
| Family XIII | 7.44 | 0.00 | 6.78 | 8.87 | 6.08 |
| Lachnospiraceae | 11.42 | 0.69 | 8.07 | 10.43 | 9.86 |
| Unclassified Lactobacillales | 3.22 | 6.77 | 2.94 | 3.66 | 1.39 |
| Microbacteriaceae | 3.87 | 3.04 | 7.02 | 5.59 | 4.11 |
| Peptococcaceae | 9.10 | 0.00 | 9.96 | 9.24 | 8.90 |
| Pseudomonadaceae | 4.62 | 4.60 | 5.29 | 3.40 | 2.77 |
| Rhizobiaceae | 1.10 | 5.50 | 8.16 | 4.52 | 4.19 |
| Ruminococcaceae | 7.95 | 0.00 | 8.22 | 9.54 | 7.11 |
| Streptococcaceae | 2.77 | 3.50 | 5.05 | 8.84 | 0.00 |
| Tannerellaceae | 2.71 | 0.00 | 4.92 | 4.34 | 6.99 |

The values for each sample correspond to the logarithmic transformation: log(x + 1), where x represents the total number of OTUs for that family in the sample.
