# Supplementary material for: Gut bacterial communities in roadkill animals: A pioneering study of two species in the Amazon region in Ecuador
Source: PLoS One. 2024 Dec 30;19(12):e0313263. doi: 10.1371/journal.pone.0313263 (PMC11684718; doi:10.1371/journal.pone.0313263)
Supplement: S6 Table — (DOCX) [file pone.0313263.s008.docx]

**Table S6. Shared OTUs among the *A. bassleri* samples correspond to the core gut microbiota.**

| OTU | Kingdom | Phylum | Class | Order | Family | Genus |
| --- | --- | --- | --- | --- | --- | --- |
| Otu00099 | Bacteria | Actinobacteria | Coriobacteriia | Coriobacteriales | Eggerthellaceae | *Eggerthella* |
| Otu00134 | Bacteria | Bacteroidetes | Bacteroidia | Bacteroidales | Bacteroidaceae | *Bacteroides* |
| Otu00095 | Bacteria | Bacteroidetes | Bacteroidia | Bacteroidales | Rikenellaceae | *Alistipes* |
| Otu00088 | Bacteria | Bacteroidetes | Bacteroidia | Bacteroidales | Tannerellaceae | *Parabacteroides* |
| Otu00184 | Bacteria | Firmicutes | Bacilli | Bacillales | Bacillaceae | *Bacillus* |
| Otu00031 | Bacteria | Firmicutes | Clostridia | Clostridiales | Christensenellaceae | *Christensenellaceae_R-7_group* |
| Otu00181 | Bacteria | Firmicutes | Clostridia | Clostridiales | Christensenellaceae | Christensenellaceae_unclassified |
| Otu00018 | Bacteria | Firmicutes | Clostridia | Clostridiales | Clostridiaceae_1 | Clostridiaceae_1_unclassified |
| Otu00009 | Bacteria | Firmicutes | Clostridia | Clostridiales | Clostridiaceae_1 | *Clostridium_sensu_stricto_1* |
| Otu00079 | Bacteria | Firmicutes | Clostridia | Clostridiales | Clostridiaceae_1 | *Clostridium_sensu_stricto_13* |
| Otu00220 | Bacteria | Firmicutes | Clostridia | Clostridiales | Clostridiales_unclassified | Clostridiales_unclassified |
| Otu01001 | Bacteria | Firmicutes | Clostridia | Clostridiales | Clostridiales_vadinBB60_group | *Clostridiales_vadinBB60_group_ge* |
| Otu00077 | Bacteria | Firmicutes | Clostridia | Clostridiales | Family_XIII | *Family_XIII_AD3011_group* |
| Otu00017 | Bacteria | Firmicutes | Clostridia | Clostridiales | Family_XIII | *Family_XIII_ge* |
| Otu00084 | Bacteria | Firmicutes | Clostridia | Clostridiales | Family_XIII | Family_XIII_unclassified |
| Otu00033 | Bacteria | Firmicutes | Clostridia | Clostridiales | Lachnospiraceae | *Epulopiscium* |
| Otu00064 | Bacteria | Firmicutes | Clostridia | Clostridiales | Lachnospiraceae | *Lachnoclostridium* |
| Otu00150 | Bacteria | Firmicutes | Clostridia | Clostridiales | Lachnospiraceae | *Lachnospiraceae_NK4A136_group* |
| Otu00007 | Bacteria | Firmicutes | Clostridia | Clostridiales | Lachnospiraceae | Lachnospiraceae_unclassified |
| Otu00056 | Bacteria | Firmicutes | Clostridia | Clostridiales | Lachnospiraceae | uncultured |
| Otu00406 | Bacteria | Firmicutes | Clostridia | Clostridiales | Peptococcaceae | Peptococcaceae_unclassified |
| Otu00180 | Bacteria | Firmicutes | Clostridia | Clostridiales | Peptococcaceae | uncultured |
| Otu00097 | Bacteria | Firmicutes | Clostridia | Clostridiales | Peptostreptococcaceae | *Paraclostridium* |
| Otu00510 | Bacteria | Firmicutes | Clostridia | Clostridiales | Peptostreptococcaceae | Peptostreptococcaceae_unclassified |
| Otu00003 | Bacteria | Firmicutes | Clostridia | Clostridiales | Peptostreptococcaceae | *Romboutsia* |
| Otu00065 | Bacteria | Firmicutes | Clostridia | Clostridiales | Peptostreptococcaceae | *Terrisporobacter* |
| Otu00129 | Bacteria | Firmicutes | Clostridia | Clostridiales | Ruminococcaceae | *Anaerotruncus* |
| Otu00472 | Bacteria | Firmicutes | Clostridia | Clostridiales | Ruminococcaceae | *Caproiciproducens* |
| Otu00131 | Bacteria | Firmicutes | Clostridia | Clostridiales | Ruminococcaceae | *GCA-900066225* |
| Otu00167 | Bacteria | Firmicutes | Clostridia | Clostridiales | Ruminococcaceae | *Oscillibacter* |
| Otu00149 | Bacteria | Firmicutes | Clostridia | Clostridiales | Ruminococcaceae | *Ruminiclostridium_5* |
| Otu00130 | Bacteria | Firmicutes | Clostridia | Clostridiales | Ruminococcaceae | *Ruminococcaceae_ge* |
| Otu00045 | Bacteria | Firmicutes | Clostridia | Clostridiales | Ruminococcaceae | *Ruminococcaceae_NK4A214_group* |
| Otu00321 | Bacteria | Firmicutes | Clostridia | Clostridiales | Ruminococcaceae | *Ruminococcaceae_UCG-013* |
| Otu00070 | Bacteria | Firmicutes | Clostridia | Clostridiales | Ruminococcaceae | *Ruminococcaceae_UCG-014* |
| Otu00093 | Bacteria | Firmicutes | Clostridia | Clostridiales | Ruminococcaceae | Ruminococcaceae_unclassified |
| Otu00363 | Bacteria | Firmicutes | Clostridia | Clostridiales | Ruminococcaceae | Uncultured |
| Otu00019 | Bacteria | Firmicutes | Erysipelotrichia | Erysipelotrichales | Erysipelotrichaceae | Erysipelotrichaceae_unclassified |
| Otu00020 | Bacteria | Firmicutes | Erysipelotrichia | Erysipelotrichales | Erysipelotrichaceae | *Turicibacter* |
| Otu00292 | Bacteria | Proteobacteria | Deltaproteobacteria | Desulfovibrionales | Desulfovibrionaceae | *Desulfovibrio* |
| Otu00032 | Bacteria | Proteobacteria | Gammaproteobacteria | Enterobacteriales | Enterobacteriaceae | Enterobacteriaceae_unclassified |
