# Supplementary material for: Gut bacterial communities in roadkill animals: A pioneering study of two species in the Amazon region in Ecuador
Source: PLoS One. 2024 Dec 30;19(12):e0313263. doi: 10.1371/journal.pone.0313263 (PMC11684718; doi:10.1371/journal.pone.0313263)
Supplement: S7 Table — (DOCX) [file pone.0313263.s009.docx]

**Table S7. Shared OTUs among the *C. ani* samples correspond to the core gut microbiota.**

| OTU | Kingdom | Phylum | Class | Order | Family | Genus |
| --- | --- | --- | --- | --- | --- | --- |
| Otu00184 | Bacteria | Firmicute | Bacilli | Bacillales | Bacillaceae | *Bacillus* |
| Otu00127 | Bacteria | Proteobacteria | Alphaproteobacteria | Rhizobiales | Beijerinckiaceae | *Methylobacterium* |
| Otu00218 | Bacteria | Proteobacteria | Gammaproteobacteria | Pseudomonadales | Pseudomonadaceae | *Pseudomonas* |
